# Supplementary material for: Comparative application of testosterone undecanoate and/or testosterone propionate in induction of benign prostatic hyperplasia in Wistar rats
Source: PLoS One. 2022 May 18;17(5):e0268695. doi: 10.1371/journal.pone.0268695 (PMC9116659; doi:10.1371/journal.pone.0268695)
Supplement: S1 Table — The levels of AST, ALT, Creatinine were measured by biochemical analyzer (AU480; Backman Coulter, Brea, CA, USA). Values are presented as means ± SD (n = 4). *p < 0.05, **p < 0.01, ***p < 0.001 compared with normal control (NC). Statistical analysis was performed by ANOVA and Dunnett’s multiple comparison test. (DOCX) [file pone.0268695.s001.docx]

**S1 Table. Organ weight and markers of liver and kidney in dosage substudy**

|  |  | Liver weight (g) | Kidney weight (g) | Spleen weight (g) | Thymus weight (g) | AST (U/L) | ALT (U/L) | Creatinine (mg/dL) |
| --- | --- | --- | --- | --- | --- | --- | --- | --- |
| NC | corn oil | 10.92±1.33 | 2.02±0.28 | 0.61±0.09 | 0.33±0.04 | 114.2±17.5 | 46.2±10.3 | 0.38±0.04 |
| CC | corn oil | 9.31±0.90 | 1.66±0.10 | 0.54±0.06 | 0.61±0.09^*^ | 81.2±5.4 | 47.2±6.14 | 0.42±0.08 |
| T undecanoate | 125mg/kg | 10.08±0.94 | 2.07±0.36 | 0.53±0.05 | 0.23±0.13 | 89.7±15.7 | 56.8±11.1 | 0.38±0.04 |
|  | 250mg/kg | 10.33±1.15 | 2.27±0.35 | 0.52±0.06 | 0.19±0.14 | 103.8±20.3 | 51.3±7.2 | 0.35±0.10 |
|  | 500mg/kg | 9.30±0.77 | 2.40±0.17 | 0.54±0.07 | 0.09±0.01 | 88.8±12.4 | 45.0±6.8 | 0.30±0.06 |
|  | 750mg/kg | 9.46±1.39 | 2.39±0.27 | 0.51±0.05 | 0.08±0.02 | 107.0±35.5 | 51.5±15.1 | 0.30±0.00 |
|  | 1000mg/kg | 9.94±0.95 | 2.46±0.37 | 0.44±0.05^**^ | 0.07±0.02 | 93.2±12.0 | 45.8±6.1 | 0.28±0.04 |
| T propionate | 125mg/kg | 9.52±1.48 | 1.81±0.21 | 0.57±0.02 | 0.53±0.14 | 77.5±6.7^*^ | 49.8±8.1 | 0.38±0.04 |
|  | 250mg/kg | 8.82±0.67 | 1.78±0.10 | 0.51±0.06 | 0.57±0.14 | 70.0±6.5^**^ | 44.2±3.7 | 0.38±0.08 |
|  | 500mg/kg | 10.27±1.80 | 1.87±0.20 | 0.55±0.10 | 0.46±0.21 | 80.3±17.6 | 57.8±15.9 | 0.40±0.09 |
|  | 750mg/kg | 10.59±0.96 | 1.88±0.21 | 0.58±0.10 | 0.42±0.10 | 95.8±22.3 | 63.2±18.4 | 0.37±0.05 |
|  | 1000mg/kg | 10.38±0.78 | 2.20±0.31 | 0.52±0.06 | 0.19±0.16 | 73.8±8.8^*^ | 52.0±6.2 | 0.35±0.05 |

The levels of AST, ALT, Creatinine were measured by biochemical analyzer (AU480; Backman Coulter, Brea, CA, USA). Values are presented as means ± SD (n = 4). ^*^p < 0.05, ^**^p < 0.01, ^***^p < 0.001 compared with normal control (NC). Statistical analysis was performed by ANOVA and Dunnett’s multiple comparison test.
