# Supplementary figures and images for: Comparative application of testosterone undecanoate and/or testosterone propionate in induction of benign prostatic hyperplasia in Wistar rats
Source: PLoS One. 2022 May 18;17(5):e0268695. doi: 10.1371/journal.pone.0268695 (PMC9116659; doi:10.1371/journal.pone.0268695)

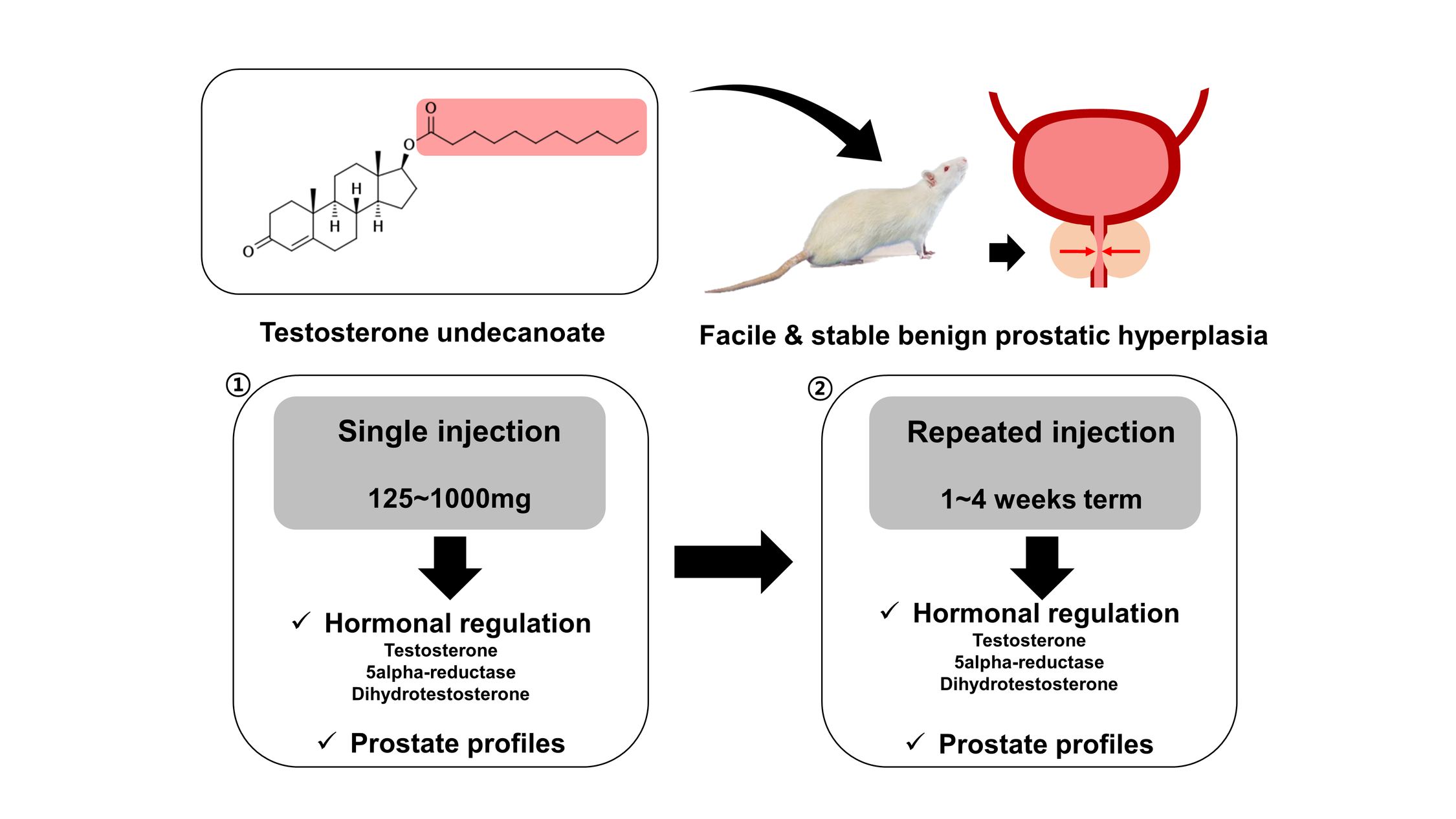

Supplement: S1 Graphical abstract — (TIF) [file pone.0268695.s003.tif]
