# Supplementary material for: Comparative application of testosterone undecanoate and/or testosterone propionate in induction of benign prostatic hyperplasia in Wistar rats
Source: PLoS One. 2022 May 18;17(5):e0268695. doi: 10.1371/journal.pone.0268695 (PMC9116659; doi:10.1371/journal.pone.0268695)
Supplement: Attachment — Submitted filename: Response to Reviewers.docx [file pone.0268695.s004.docx]

**This is our opinion in response to the reviewer’s comments on our manuscript, PONE-D-21-40865. In the revised manuscript, we had carefully examined our manuscript again and tried our best to clear the issues raised by the reviewers.**

Reviewer #1: Dear Authors,

Thank you for the opportunity to review your manuscript titled, "Stable Hormone Regulation using Testosterone Undecanoate for Inducing Benign Prostatic Hyperplasia in Wistar Rats ". The topic is interesting and of great importance. The authors determined the optimal dosage and dosing cycle using testosterone undecanoate for efficient and stable induction of benign prostatic hyperplasia than testosterone propionate. The manuscript is well written. There are certain issues that the authors need to address before the paper can be accepted for publication and I hope to be useful for improving the manuscript.

1- Please, titles and subtitles of manuscript parts should follow the instruction of the journal.
Title
- We had carefully check again and it was modified. (line 1-2)

2- The authors compared the using of testosterone undecanoate and / or testosterone propionate in induction of benign prostatic hyperplasia in all manuscript. So please mention this in your title. I suggest "Comparative application of testosterone undecanoate and / or testosterone propionate in induction of benign prostatic hyperplasia in Wistar rats"
-We appreciate your suggestion.We changed the title following your suggestion. (line 1-2)

Abstract
3- Please rewrite the method section determining the period of examining in both studies (8 and 12 weeks).
- The period of examining in both studies was inserted in the method section. (line 22, 24)

Introduction
4- Introduction is well written.
5- Please insert the full name beside the abbreviation if mentioned for first time, and please unite the abbreviations in all manuscript. Example. Testosterone undecanoate (T undecanoate), testosterone propionate (T propionate) and wrote in tables footnotes of table 3…(TU), (TP).
Materials and methods
-The abbreviations in manuscreipt were checked and modified. (line 43, 51, 70, 246-248)

6- Please insert feeding and drinking regime of rats during experiment period.
- The feeding and drinking regime were inserted in method. (line 93)

7- Please determine exact time of hormonal injection after castration.
- We modified the procedure of horemanal injection in method. (line 93-95)

8- Please insert dose of anesthetic agents used in castration of rats with reference.
- The dose of anesthetic agents was inserted in method. (line 97)

9- The authors wrote "Each group consisted of 5–6 rats at the beginning of the experiment, total 69 rats were used" in dosage sub study section. Please determine the exact number of rats used in each group during dosage sub study and rewrite it in this study ' tables footnotes.
- The exact number of rats used in each group during dosage sub study rewrited in method and tables footnotes. (line 107-108)

10- Please replace subtitle in line 112 " Androgens and 5-alpha reductase type 2" to "Blood sampling and hormonal analysis"
- We changed the title following your suggestion. (line 116)

11- The authors wrote in statistical analysis section " Data are presented as the mean ± standard error of the mean (SEM)" and wrote in tables footnotes and figures legends "Standard deviation (SD)". Please check.
- Statistical analysis section was checked and modified. (line 141)

Results
12- Please use or remove the abbreviations in footnotes of table 3 concerning prostatic weight, volume and index in table 3.
- The abbreviations in footnotes of table prostatic weight, volume and index was removed. (line 248-249)

13- Please insert arrows on figures of histopathology to clarifying the major differences between groups especially "epithelial thickness of the prostate tissue".
-The arrows on figures of histopathology were inseted. (Figure 3, 4)

14- The authors not mentioned the parameters mentioned in supplementary (supporting) information except only in tables S1, S2 and in discussion section lines 290-292. Please check and add in details or remove.
- Explanation of the parameters in S1 was added in ditails and S2 was removed. (line 188-190)

Discussion
15- It needs more explanations for your results with needs of previous literatures that agree and disagree with your results.
- The explanation of comparison between our results and previous literatures was modified. (line 313-314)

Reviewer #2: This is the first study on application of long-acting potential of T undecanoate to induce BPH instead to treat gonadal function by considering optimal dosage and dosing cycle. It showed the extended application of T undecanoate for induction of BPH, with advantages of maintenance that is more convenient and stable disease marker than inducing BPH using T propionate.
-We appreciate your review.

Reviewer #3: Comments to the authors:
This manuscript entitled “Stable Hormone Regulation using Testosterone Undecanoate for Inducing Benign Prostatic Hyperplasia in Wistar Rats”. The manuscript is describing mainly the optimal dosage and dosing cycle for efficient and stable induction of benign prostatic hyperplasiausing using testosterone undecanoate. The injection cycle substudy, testosterone undecanoate-administered group stably maintained high levels of testosterone, dihydrotestosterone, and 5-alpha reductase compared with testosterone propionate-administered group for the same injection cycl. Some other points should be take in consideration for the improvement of the manuscript. The title needs much more modifications to match the designe. The manuscript is organized ,informative and well presented but needs some modifications.
- We appreciate your review and totally agree with your opinion. The title was modified to match the designe. (line 1-2)

Firistly , There were some notes
- The T undecanoate : should be mentioned in the first appearance as full term , then the abbreviation within the whole manuscript.
-The abbreviations in manuscreipt were checked and modified. (line 51)

- Mention the most recent studies on prostatic hyperplasia causes , consequences and in-vivo models.
-The reference of most recent studies on prostatic hyperplasia causes , consequences and in-vivo models was added. (line 55-58)

- The clinical uses and trials on exogenous testosterone should be updated and its clinical side effects.
- The clinical uses, trials and side effects on exogenous testosterone was updated (line 48-50)

- The rationale of selected doses for the treatment must be explained
- The explantaion of rationale of selected doses for the treatment was modified. (line 106)

- The attribution of the histopasthological alterations are not well described in the discussion , revise
- The attribution of the histopasthological alterations was added in discussion. (line 294-297)

- The conclusion needs to be rewritten in accordance to the aim and hypothesis of the study
- The conclusion was modified in accordance to the aim and hypothesis of the study. (line 322-324)
