# Supplementary material for: Comparative application of testosterone undecanoate and/or testosterone propionate in induction of benign prostatic hyperplasia in Wistar rats
Source: PLoS One. 2022 May 18;17(5):e0268695. doi: 10.1371/journal.pone.0268695 (PMC9116659; doi:10.1371/journal.pone.0268695)
Supplement: Attachment — Submitted filename: Response to Reviewers.docx [file pone.0268695.s005.docx]

**This is our opinion in response to the reviewer’s comments on our manuscript, PONE-D-21-40865. In the revised manuscript, we had carefully examined our manuscript again and tried our best to clear the issues raised by the reviewers.**

Reviewer #1: Dear Authors,

Thank you for the opportunity to review your manuscript titled, "Stable Hormone Regulation using Testosterone Undecanoate for Inducing Benign Prostatic Hyperplasia in Wistar Rats ". The topic is interesting and of great importance. The authors determined the optimal dosage and dosing cycle using testosterone undecanoate for efficient and stable induction of benign prostatic hyperplasia than testosterone propionate. The manuscript is well written. There are few issues that the authors need to address and I hope to be useful for improving the manuscript.

1- Please, titles and subtitles of manuscript parts should follow the instruction of the journal.

- The instruction of the journal fo titles and subtitles of manuscript was check again.

Introduction

2- Please insert the full name beside the abbreviation if mentioned for first time, and please unite the abbreviations in all manuscript. Example BPH in page 3, line 49 and full name was mentioned in line 53. Also, WHO and FDA in lines 46, 47. Please check.

- The full name beside the abbreviation at the first time of mention was modified.(at line 46-47, 49, 54)

Results

The authors not mentioned the parameters mentioned in supplementary (supporting) information except only in tables S1. Please check and add in details or remove.

- The parameters of supplementary (supporting) information modified more detail.(at line 190-192)

Reviewer #3: The authors addressed most of the required points but still major concerns about the rationale for the selected doses and also the hypothesis of the current study. In adddition the discussion still needs substantial revision especially regarding the results of histopathology especially of the prostate gland ( attributions and recent citations).

- The discusstion of the rationale for the selected doses and the hypothesis of the current study were modified in detail. (at line 278-279, ) And, the discussions of the histopathology of the prostate gland and the hypothesis were modified. (at line 298-301)

There is a nother major concern , Why the authors deleted the acknowlegment which is presented in the first copy of the manuscript. any concerns regarding this must be disclosed in a seperate file and disclose by all athors.

- The acknowledgment, which presented in the first copy of the manuscript, was modified as directed by Journal Requirements (Journal Requirements: Please remove any funding-related text from the manuscript and let us know how you would like to update your Funding Statement.) in first revision. And, the acknowledgment content was included in the Funding Statement section of the online submission form.
