# Supplementary material for: Comparative application of testosterone undecanoate and/or testosterone propionate in induction of benign prostatic hyperplasia in Wistar rats
Source: PLoS One. 2022 May 18;17(5):e0268695. doi: 10.1371/journal.pone.0268695 (PMC9116659; doi:10.1371/journal.pone.0268695)
Supplement: Attachment — Submitted filename: Response to Reviewers.docx [file pone.0268695.s006.docx]

**This is our opinion in response to the reviewer’s comments on our manuscript, PONE-D-21-40865R2. In the revised manuscript, we had** **carefully examined our manuscript again and tried our best to clear the issues raised by the reviewers.**

Journal Requirements:

Please review your reference list to ensure that it is complete and correct. If you have cited papers that have been retracted, please include the rationale for doing so in the manuscript text, or remove these references and replace them with relevant current references. Any changes to the reference list should be mentioned in the rebuttal letter that accompanies your revised manuscript. If you need to cite a retracted article, indicate the article’s retracted status in the References list and also include a citation and full reference for the retraction notice.

-The reference list was carefully reviewed and modified (at line 339-341, 361, 363, 367, 371)

Reviewer #1

Please insert the methods of measuring of the parameters mentioned in supplementary (supporting) information.

- The methods of measuring of the parameters in supplementary (supporting) information was added (at line 419)
